# Epidemiology of Herpes Zoster in the pre-vaccination era: establishing the baseline for vaccination programme’s impact in Spain

**DOI:** 10.2807/1560-7917.ES.2023.28.8.2200390

**Published:** 2023-02-23

**Authors:** Carlos Risco Risco, Zaida Herrador, Noemí Lopez-Parea, Diego Martínez-Urbistondo, Rafael Suárez del Villar Carrero, Josefa Masa-Calles

**Affiliations:** 1 Internal Medicine Service, Hospital HM Sanchinarro, Madrid, Spain; 2 National Centre of Epidemiology, Carlos III Institute of Health, Madrid, Spain; 3 CIBER in Epidemiology and Public Health (CIBERESP), Madrid, Spain

**Keywords:** Herpes Zoster, Herpes Zoster Vaccine, Epidemiology, Spain

## Abstract

**Background:**

Herpes zoster (HZ) affects 1 in 3 persons in their lifetime, and the risk of HZ increases with increasing age and the presence of immunocompromising conditions. In Spain, vaccination guidelines were recently updated to include the recommendation of the new recombinant zoster vaccine (RZV) for certain risk groups.

**Aim:**

To describe the epidemiology of HZ-related hospitalisations in Spain in order to prioritise vaccination recommendations and define a baseline to monitor the effectiveness of vaccination policies.

**Methods:**

Retrospective study using the National Health System’s Hospital Discharge Records Database, including all HZ-related hospitalisations from 1998 to 2018.

**Results:**

The 65,401 HZ-related hospitalisations, corresponded to an annual mean hospitalisation rate of 6.75 per 100,000 population. There was an increasing trend of HZ hospitalisations over the study period. This rate was higher in males and older age groups, particularly over 65 years. Comorbidities with higher risk of readmission were leukaemia/lymphoma (RR 2.4; 95% CI: 2.3–2.6) and solid malignant neoplasm (RR 2.2; 95% CI: 2.1–2.4). Comorbidities associated with higher risk of mortality were leukaemia/lymphoma (RR 2.9; 95% CI: 2.7–3.2), solid malignant neoplasm (RR 2.9; 95% CI: 2.7–3.1) and HIV infection (RR 2.2; 95% CI: 1.8–2.7).

**Conclusion:**

Of all patients hospitalised with HZ, those with greater risk of mortality or readmission belonged to the groups prioritised by the current vaccination recommendations of the Spanish Ministry of Health. Our study provided relevant information on clinical aspects of HZ and established the base for future assessments of vaccination policies.

**Key public health message**

**What did you want to address in this study?**

We wished to describe the epidemiology of herpes zoster (HZ, also known as shingles)-related hospital admissions and assess the severity of HZ in Spain. This information is necessary to establish a baseline for comparison and to judge if a national vaccination programme with a new recombinant vaccine is effective.

**What have we learnt from this study?**

Herpes zoster is an increasing health problem in Spain and it is associated with ageing and severity in certain risk groups. People with conditions that adversely affect their immune system's defences, such HIV infection, leukaemia, lymphoma or solid malignant neoplasm, as well as people older than 65 years have greater risk of severe HZ.

**What are the implications of your findings for public health?**

Efforts should be made to reach groups who have been prioritised to receive the HZ vaccine. Since adequate vaccination coverage in adults is usually difficult to reach, communication activities, recommendations and advice from healthcare professionals involved in vaccination activities should be conducted to reach these groups.

## Introduction

Varicella zoster virus (VZV) is a human neurotropic herpes virus which causes two distinct diseases that can be prevented by vaccines. Varicella, the primary infection usually occurs in childhood, and herpes zoster (HZ), also called shingles, is the result of the reactivation of latent VZV. Herpes zoster is characterised by a vesicular skin rash localised in the dermatomes correspondent to the affected ganglia, and is often preceded, or accompanied, by acute neuropathic pain or itching. The most common complication of HZ is post-herpetic neuralgia (PHN), operationally defined as pain lasting at least 90 days after rash onset and impacting the patients' quality of life. Other frequent complications include brain inflammation, pneumonia, hearing problems and complications involving the eye. These complications may happen simultaneously [1,2].

More than 90% of the world population harbours a latent VZ virus, of whom more than 50% will experience a reactivation by 85 years of age [3]. A median HZ incidence of 6.6–9.03 per 1,000 person-years has been estimated for North America, 5.23–10.9 for Europe and 10.9 for the Asia-Pacific region [4]. Incidence rates of HZ increase with age, particularly in those over 50 years old [5,6]. Post-herpetic neuralgia has also been seen to increase in persons older than 50 years of age with HZ, occurring in 18% of persons over 50 years old and 33% of persons over 80 years old [5]. The cumulative incidence of HZ seems to be increasing over time globally [4].

The incidence of HZ increases with age and is related to waning specific cell-mediated immunity (CMI). Severely immunocompromising conditions such as leukaemia, lymphoma, stem cell and solid organ transplant, HIV infection and AIDS have been largely associated with greater risk of HZ [7]. Other chronic conditions such as rheumatoid arthritis, inflammatory bowel disease, asthma, chronic obstructive pulmonary disease, ischemic heart disease, corticoids, Janus kinase (JAK) inhibitors exposure, and diabetes mellitus type 1 are considered of moderate risk for herpes zoster [8]. Despite individuals with immunocompromising conditions representing a small fraction of overall HZ cases (around 8% in the United States (US)) [5], their condition constitutes a healthcare burden because of the higher risk for HZ complications, including HZ ophthalmicus and PHN, and related mortality [8].

The clinical relevance of HZ and its increased incidence has prompted a search for more effective prevention measures and therapies, with a number of vaccines having been developed in recent years [9]. So far, two vaccines against HZ and its complications, particularly PHN, have been licensed for adults: a live attenuated VZV vaccine (HZ vaccine live (ZVL), Zostavax, Merck, US) and an adjuvanted VZV glycoprotein E (gE) sub-unit vaccine (HZ/su) (recombinant HZ vaccine (RZV), Shingrix, GlaxoSmithKline, United Kingdom). Both vaccines have been proven safe and effective in reducing the incidence and severity of HZ, while RZV seems to be more effective for the prevention of HZ [10,11]. In Spain, since 2018 HZ vaccination with HZ/su has been recommended for adults with certain immunocompromising conditions. Guidelines were updated in 2021 prioritising vaccination for certain groups. Vaccination is progressively incorporated in the Spanish population, with those between 65 and 80 years of age vaccinated first [12]. Further information on the vaccination strategy and catch up is provided in the Box.

Box **National recommendations for HZ/su immunocompromising conditions vaccination in Spain, 2021 [11]**

1) To incorporate the recombinant herpes zoster vaccination (RZV) in people over 18 years of age with the following risk conditions, as soon as enough doses are available:

(i) Haematopoietic stem cell transplantation. Requires 2 doses administered at an interval of a least 2 months:

(ii) Solid organ transplant. Requires 2 doses administered at an interval of at least 2 months;

(iii) Treatment with anti Janus kinase inhibitor (JAK) drugs. Requires 2 doses administered at an interval of at least 2 months, before starting treatment if possible;

(iv) HIV. Vaccination will be performed in persons on antiretroviral therapy with a stable clinical situation for at least 1 year. Two doses will be given 2 months apart;

(v) Malignant blood diseases. Requires 2 doses administered at an interval of at least 2 months. In the case of persons starting chemotherapy treatment, the first dose should be administered at least 10 days before starting the first cycle of treatment;

(vi) Solid tumours being treated with chemotherapy. Requires 2 doses administered at an interval of at least 2 months. The first dose may be given after completing the course of antitumour therapy or look for window periods for its administration.

2) Following confirmation of vaccine availability, incorporate routine RZV in the 65-year-oldcohort in 2022. Depending on its availability, at least one cohort per year will be vaccinated, starting with the cohort turning 80 years of age and decreasing in age until reaching the first cohort to be vaccinated at 65 years. People who have previously received the varicella vaccination (VV) may be vaccinated by administering the first dose of RZV at least 5 years after VV.

In Spain, HZ has been a mandatory notifiable disease since 2007, with aggregated information on sex, age group and varicella vaccination status to be reported once a year. However, no information about underlying conditions, hospitalisations or death in HZ cases can be extracted from this data source [12]. In addition to this case notification system, severity tracking of HZ can be estimated through the analysis of the National Health System’s Hospital Discharge Records Database (CMBD). Using the CMBD, we aim to describe the epidemiology of HZ hospitalisations in Spain from 1998 to 2018 in order to establish a baseline for future measurements of the vaccination programme’s impact (Box).

## Methods

### Study design

Using the Spanish CMBD, we performed a retrospective descriptive study of HZ epidemiology between 1 January 1998 and 31 December 2018.

### Data source

The CMBD is part of Spain’s Health Information System. It is a set of mandatory and anonymised standardised clinical-administrative data of hospitalisations that provides information on patient morbidity in public and private hospitals. It is estimated to cover 99.5% of the Spanish population. In 2016, the Register of Specialised Health Care Activity (RAE-CMBD) was implemented as a new data model. In both CMBD and RAE-CMBD, up to 20 diagnostic variables are recorded, corresponding to diagnostics performed during hospitalisation. International Classification of Diseases, Ninth Revision, Clinical Modification (ICD-9-CM) for 1998–2015 and Tenth Revision Clinical Modification (ICD-10-CM) for 2016–2018 were used [13]. In this study, any discharge with ICD-9-CM codes 053.0-053.9 or ICD-10-CM codes B02.0-B02.1 or B02.30-B02.39 for HZ in any of the possible diagnostic fields were selected [13]. Readmissions were not excluded as we aimed to assess hospitalisations rather than patients.

For each entry, socio-demographic (sex as a binary variable, age and autonomous community of residency) and clinical data (date of admission, HZ complications, other related diagnoses, re-admission, death) were extracted from the CMBD dataset. Hospital readmission is defined as when a patient who had been discharged from a hospital is admitted again within 30 days after discharge.

Herpes zoster complicated forms included: external otitis; ophthalmological complications; central nervous system HZ or HZ meningitis/encephalitis; HZ post herpetic neuralgia; and disseminated HZ. Underlying and related clinical conditions for HZ cases were assessed by searching for all diagnostic codes in any diagnostic position. We extracted the following comorbidities and conditions related to HZ in line with the medical literature: diabetes mellitus (DM); rheumatologic disorders; chronic obstructive pulmonary disease (COPD); chronic kidney disease; HIV infection; solid organ transplantation; haematopoietic progenitor cell transplantation; leukaemia or lymphoma; and solid malignant neoplasm (see ICD-9-CM and ICD-10-CM in Supplementary Table S1).

Official population figures from the National Statistics Institute (Instituto Nacional de Estadística in Spanish (INE)) [14] were used as ‘population at risk’ for the study period.

### Data analysis

Age was categorised into five groups: ≤ 24 years, 25–44 years, 45–64 years, 65–84 years and ≥ 85 years. Frequencies, percentages, means and standard deviations (SD), medians and interquartile range (IQR) were used to summarise data. Differences in proportions were assessed using the chi-squared test. The student t-test was used to compare differences in the means.

The average number of HZ-related hospitalisations per year and region were calculated to assess temporal and geographical patterns. Mean hospitalisation rates were plotted in maps using the Geographical Information System QGis [15]. Temporal trends in hospitalisation rates for the whole population studied and by age group were computed using linear regression analysis.

To assess the association between sociodemographic and clinical characteristics with readmission and fatal outcome, modified Poisson regression models were obtained using a manual backward stepwise procedure. Risk ratios (RR) were computed with 95% confidence intervals (CI). Age, sex and other comorbidities were tested as adjustment variables and p values < 0.05 were considered statistically significant. Data analysis was performed using STATA software version 16.1 (StataCorp LLc, College Station, US).

## Results

### Socio-demographic and clinical characteristics

Of the 65,401 hospitalised patients with HZ, 51.2% (n = 33,464) were male and 48.8% (n = 31,937). The mean age was 65.9 years, with a median of 71 years (interquartile range (IQR): 56–81). Median age was slightly higher in hospitalised women (74 and 70 in women and men, respectively, p < 0.05). The age groups above 65 years old were the most represented (63.7%). The median length of stay was 8 days (IQR: 5–14), and 14.7% of HZ-related hospitalisations were readmissions during the first month after discharge (information only available until 2015). Males were more frequently readmitted (p < 0.01). Death occurred in 5.8% of all hospitalisations. Concerning those comorbidities most frequently related to HZ in the literature, DM (19.2%) and leukaemia/lymphoma (14.1%) were the most prevalent ones among HZ-related hospitalisations in Spain during the study period. Comorbidities were unequally distributed by sex (Table 1).

**Table 1:** Sociodemographic and clinical characteristics of herpes zoster-related hospitalisations, Spain, 1998–2018 (n = 65,401)

Characteristics		Males (n = 33,464)		Females (n = 31,937)		p value
		n	%	n	%	
**Age group (years)**	**0–24**	1,989	53.87	1,703	46.13	< 0.001
	**25–44**	3,796	56.61	2,910	43.39	
	**45–64**	7,457	55.78	5,912	44.22	
	**65–84**	16,435	52.30	14,992	47.70	
	**≥ 85**	3,787	37.10	6,420	62.90	
**Length of stay (days)**	**< 7**	12,148	50.67	11,829	49.33	0.051
	**≥ 7**	21,136	51.46	20,108	48.54	
**Readmission in first month after discharge^a^**	**Yes**	4,553	58.41	3,242	41.59	< 0.001
	**No**	22,799	50.60	22,259	49.50	
**Type of hospital discharge**	**Home**	819	49.01	852	50.99	0.002
	**Transfer**	863	52.98	766	47.02	
	**Death**	2,035	53.61	1,761	46.39	
	**Other**	819	49.01	852	50.99	
**Comorbilidities^b^**	**Diabetes mellitus**	6,260	18.71	6,281	19.67	< 0.001
	**Asthma**	578	1.73	1,711	5.36	
	**Rheumatologic diseases**	942	2.81	1,471	4.61	
	**Chronic obstructive pulmonary disease**	5,021	15.00	1,349	4.22	
	**Chronic kidney disease**	2,624	7.84	2,288	7.16	
	**HIV infection**	2,049	6.12	975	3.05	
	**Solid organ transplantation**	1,244	3.78	832	2.64	
	**Haematopoietic transplantation**	511	1.53	391	1.22	
	**Leukaemia or lymphoma**	5,229	15.63	4,020	12.59	
	**Solid malignant neoplasm**	2,504	7.48	1,713	40.77	

^a^ Data only available from 1998 to 2015.

^b^ Several comorbidities may exist in the same patient.

Most hospitalisations were related to non-complicated HZ (77.1%). External otitis (25.1%) and ophthalmological complications (11.1%) were also common. Non-complicated HZ, external otitis and disseminated HZ were significantly more common among older age groups (p < 0.05), while HZ ophthalmological complications, Central Nervous System (CNS) infections/other CNS complication and post herpetic neuralgia were more frequent among younger age groups (p < 0.05) (Table 2).

**Table 2:** Complications by age group among herpes zoster-related hospitalisations, Spain, 1998–2018 (n = 65,401)

Clinical presentation of herpes zostera	Age group (years)										Total	
	0–24		25–44		45–64		65–84		≥ 85			
	n	%	n	%	n	%	n	%	n	%	n	%
**Non-complicated**	**2,538**	**68.74**	**4,797**	**71.53**	**10,252**	**76.68**	**24,729**	**78.69**	**8,111**	**79.47**	**50,427**	**77.10**
**External otitis**	588	15.93	1,360	20.28	3,346	25.03	8,150	25.93	2,937	28.77	16,381	25.05
**Ophtalmological complications**	500	13.54	790	11.78	1,389	10.39	3,485	11.09	1,092	10.70	7,256	11.09
**CNS infection /other CNS complication**	252	6.83	511	7.62	670	5.01	1,403	4.46	373	3.65	3,209	4.91
**Postherpetic neuralgia**	281	7.61	511	7.62	524	3.92	958	3.05	251	2.46	2,525	3.86
**Diseminated herpes zoster**	91	2.46	178	2.65	401	3.00	782	2.49	317	3.11	1,769	2.70

CNS: central nervous system.

^a^ Several clinical forms and comorbidities might exist in the same patient.

### Temporal and spatial trends in Spain

Between 1998 and 2018, there were 65,401 hospitalisations with diagnosis of HZ in any diagnostic position recorded in the RAE-CMBD database. The ICD-9 or ICD-10 codes were positioned as a first diagnosis in 17,977 records (27.5%) and as a second diagnosis in 23,635 records (36.1%).

**Figure 1:**
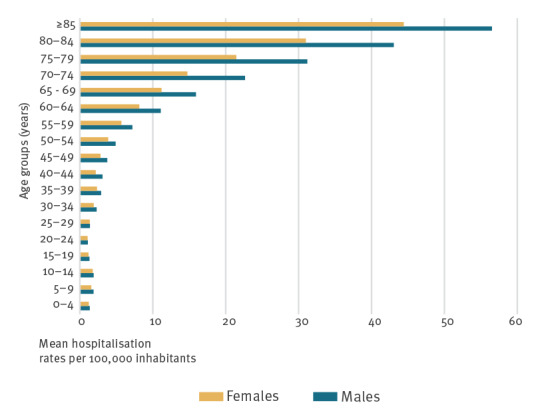
Herpes zoster annual hospitalisation rates by sex and age group, Spain, 1998–2018 (n = 65,401)

The mean HZ hospitalisation rate for the period of study was 6.75 per 100,000 population (ranging from 5.82 in 1998 to 9.21 in 2018) with an increasing trend over the study period (Figure 1). When comparing mean HZ hospitalisation rates by age group, relevant differences emerged: overall, the age group ≥ 85 years showed the highest rate (46.40 per 100,000) for the whole study period, followed by those 65–84 years old (22.16 per 100,000). The youngest age groups showed the lowest hospitalisation rates (1.50 under 25 years old and 2.30 for those25–44 years old). Annual hospitalisation rates were higher in males in all age groups during the study period but were particularly greater in those over 65 years old (Figure 1).

**Figure 2:**
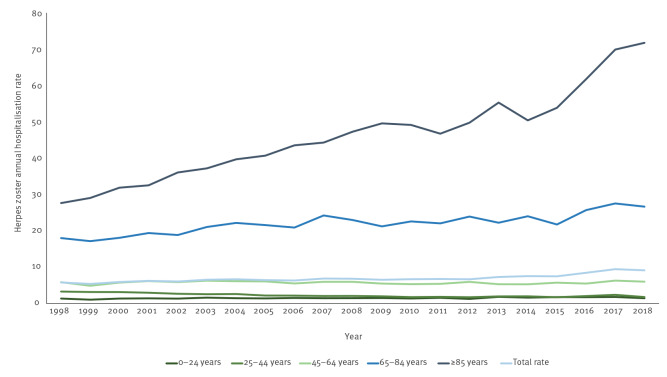
Temporal trends of herpes zoster annual hospitalisation rates by age group, Spain, 1998–2018 (n = 65,401)

Regarding temporal trends, we found an overall significant increasing trend in hospitalisation rates from 1998 to 2018 (p < 0.05). The annual hospitalisation trend decreased for the 25–44 age group (p < 0.01) and increased for all others except for the 45–64 age group, which showed a stable pattern over the study period. The major rising trend in annual hospitalisation rates was observed among people over 85 years (p *<* 0.01) (Figure 2).

**Figure 3:**
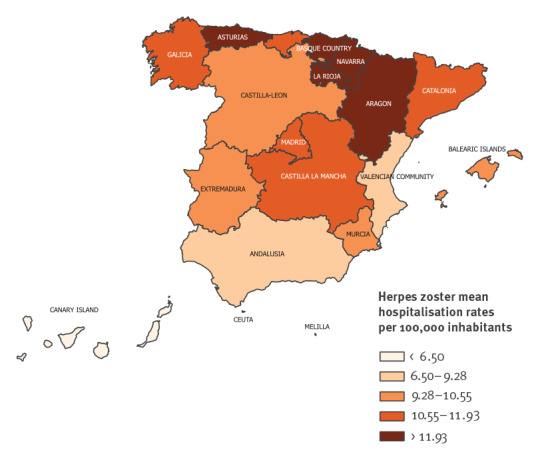
Herpes zoster mean hospitalisation per 100,000 inhabitants by autonomous community, Spain, 1998–2018 (n = 65,401)

Regarding regional distribution, mean HZ hospitalisation rates followed a north-south gradient: Asturias, Basque Country, Navarra, La Rioja and Aragón had the highest mean HZ hospitalisation rates (over 11.9 hospitalisations per 100,000 population). Mean hospitalisation rates were below 9.3 in Andalusia, Canary Islands, Valencian Community and the autonomous cities of Ceuta and Melilla (Figure 3).

### Risk factors for readmission and death

Males were more prone to being readmitted to hospital than females, although this association decreased after adjusting for comorbidities (adjusted relative risk (aRR): 1.3, 95% CI: 1.1–1.4 and aRR: 1.2, 95% CI: 1.1–1.2, respectively). The risk of being readmitted decreased with age. All comorbidities were associated with an increased risk of readmission. Leukaemia/lymphoma and solid malignant neoplasm had the highest aRR of all comorbidities (2.4, 95% CI: 2.3–2.6 and 2.2, 95% CI: 2.1–2.4, respectively) (Table 3).

**Table 3:** Factors related to readmission and fatal outcome in herpes zoster hospitalisations, Spain 1998–2018 (n = 65,401)

Variables		Factors related to re-admission							Factors related to fatal outcome					
		n	%	RR adjusted for sex and age group		RR adjusted for sex, age, and comorbidities			n	%	RR adjusted for sex and age group		RR adjusted for sex, age, and comorbidities	
				RR	95% CI	RR		95% CI			RR	95% CI	RR	95% CI
**Sex**	**Females**	3,243	12.71	**1**	**NA**	**1**	**NA**		1,761	5.51	1	NA	1	NA
	**Males**	4,553	16.65	1.31	1.26–1.37^a^	1.16	1.12–1.22^a^		2,035	6.08	1.1	1.04–1.17^b^	1.24	1.20–1.28^a^
**Age group (years)**	**0–24**	648	20.79	1	NA	1	NA		40	1.08	1	NA	1	NA
	**25–44**	770	13.06	0.63	0.57–0.69^a^	0.69	0.63–0.77^a^		152	2.27	2.09	1.48–2.96^a^	0.72	0.65–0.79^a^
	**45–64**	1,850	16.77	0.81	0.74–0.87^a^	0.75	0.70–0.81^a^		568	4.25	3.92	2.85–5.39^a^	0.93	0.86–1.01
	**65–84**	3,799	14.90	0.72	0.67–0.77^a^	0.72	0.67–0.77^a^		1,950	6.20	5.73	4.20–7.82^a^	0.94	0.88–1.02
	**≥ 85**	728	9.96	0.48	0.43–0.53^a^	0.57	0.51–0.63^a^		1,086	10.64	9.82	7.18–13.44^a^	0.92	0.85–0.99^b^
**Comorbidities**	**Diabetes mellitus**	1,445	14.86	1.04	0.99–1.10	1.12	1.07–1.19^a^		736	5.87	0.85	0.79–0.92^a^	0.88	0.82-.0.95^b^
	**Asthma**	288	15.64	1.15	1.03–1.28^b^	1.38	1.24–1.54^a^		109	4.76	0.82	0.68–0.99^b^	0.92	0.77–1.11
	**Rheumatologic diseases**	290	16.36	1.12	1.01–1.25^a^	1.21	1.09–1.35^a^		149	6.17	1.10	0.94–1.29	1.18	1.01–1.38^b^
	**Chronic obstructive pulmonary disease**	1,041	21.67	1.51	1.42–1.60^a^	1.76	1.66–1.87^a^		421	6.61	0.94	0.85–1.04	1.02	0.92–1.12
	**Chronic kidney disease**	546	18.33	1.36	1.25–1.47^a^	1.40	1.29–1.52^a^		465	9.47	1.32	1.20–1.45^a^	1.39	1.27–1.53^a^
	**HIV infection**	410	14.62	0.99	0.90–1.11	1.11	1.02–1.22^a^		121	4.00	1.48	1.22–1.79^a^	2.22	1.83–2.69^a^
	**Solid organ transplantation**	404	21.7	1.46	1.34–1.60^a^	1.65	1.51–1.88^a^		67	3.23	0.69	0.66–0.88^b^	0.80	0.63–1.01
	**Haematopoietic transplantation**	158	20.76	1.23	1.07–1.42^b^	NA	NA		26	2.88	0.88	0.6–1.29	NA	NA
	**Leukaemia or lymphoma**	2,197	26.94	2.03	1.94–2.12^a^	2.45	2.34–2.57^a^		937	10.13	2.41	2.25–2.89^a^	2.95	2.74–3.17^a^
	**Solid malignant neoplasm**	15	30	1.72	1.62–1.83^a^	2.22	2.09–2.37^a^		515	12.36	2.31	2.13–2.51^a^	2.89	2.66–3.15^a^

CI: confidence interval; NA: not applicable; RR: risk ratio.

^a^ p < 0.001.

^b^ p < 0.005.

Male sex was significantly associated with higher mortality during hospitalisation (aRR: 1.2, 95% CI: 1.2–1.3). There was an interaction between age and the presence of comorbidities; risk of death increased with age when comorbidities were not included in the multivariate analysis, but this relationship reversed for all age groups when adjusting for comorbidities. Comorbidities significantly associated with higher mortality risk were leukaemia/lymphoma (aRR: 2.9, 95% CI: 2.7–3.2), solid malignant neoplasm (aRR: 2.9, 95% CI: 2.7–3.1) and HIV infection (aRR: 2.2, 95% CI: 1.8–2.7) (Table 3).

## Discussion

This analysis provides a 21-year review of HZ-related hospitalisations and risk factors for readmission and fatal outcome in Spain. Currently, there are two vaccines against HZ and its complications available, one live attenuated VZV vaccine (ZVL)), and a more recent recombinant HZ vaccine (RZV). Although immunosuppression is associated with a high burden of HZ and its complications, ZVL is contraindicated in immunocompromised individuals as it may cause VZV-related disease [16]. Because of this, the Spanish Ministry of Health did not consider implementing the HZ vaccination until 2018, when the Interterritorial Council of the National Health System (CISNS in Spanish) recommended vaccinating with RZV for concrete risk groups. However, this was not applied initially due to the lack of vaccines. In March 2021, the guidelines were revised and updated (see concise summary). Progressively, and depending on vaccine availability, routine vaccination is being incorporated for the general population above 65 years of age [11,17]. Our results shed light on the occurrence of HZ and the magnitude of this public health problem in Spain and help establish the baseline for future vaccine evaluations. Factors possibly influencing the future evolution of HZ are the natural history of the disease, the availability of RZV vaccines and other intermediate variables such as universal varicella vaccination, population ageing or the increasing prevalence of chronic conditions and intake of immunosuppressive treatments.

In consonance with our data, greater annual hospitalisation numbers in elderly people have been widely described [18]. Regarding sex differences, hospitalisation rates were higher in males than in females in all age groups. On the other hand, according to Spanish surveillance data, HZ global incidence was higher in females [17,19]. In a recent systematic review, all studies reported a higher incidence of HZ in females compared with males, except in the highest age groups [4]. This disparity may account for certain interaction with age and other factors related to severity and clinical characteristics in males, which may act as confounders [2,20,21].

Fatal outcome occurred in 5.8% of HZ-related hospitalisations in our data. A European systematic review showed that in hospital case fatality rates vary from 0.4% in those aged 60–69 years in Portugal, to 7.1% in those aged 80 years old and older in Spain [2].

The overall hospitalisation rate for the study period was 6.75 per 100,000 population. According to previous research based on surveillance data, between 2014 and 2018, the community mean incidence of HZ in Spain was around 351.6 per 100,000 inhabitants, almost doubling in those over 50 years old (625.5) [17]. Similarly, in other countries it has been observed that hospitalisation occurs in barely 1% of the affected population, and mainly in the oldest age groups [22,23]. Our hospitalisation rates were much higher at older ages. Evidence shows that older age is the greatest risk factor for HZ reactivation, mainly due to decline in cellular immunity, increasing prevalence of chronic diseases and growing use of immunosupressant medication [20,24].

We also observed an increasing temporal trend during the study period, especially in the age groups over 65 years. In other countries, HZ hospitalisation rates also showed an increasing trend [21,25] or remained steady [6] in the last few decades. On the contrary, in the US where the administration of HZ vaccine has been recommended for people over 60 years since 2008, a decline in HZ hospitalisations has been observed, despite a low vaccination coverage [26]. Several countries have also reported that HZ hospitalisations have decreased after implementation of varicella vaccination [27-29]. On the other hand, it has been argued that universal varicella vaccination programs could alter the epidemiology of HZ due to a reduced circulation of VZV among children, which would weaken the population immunity against VZV resulting in an increase in HZ cases [29,30]. Therefore, the impact that childhood vaccination of varicella may have on the epidemiology of HZ remains a controversial issue and may require decades of further research.

Regarding regional distribution, a north-south gradient was observed in our study. These spatial results should be taken with caution; the regional distribution of immunocompromising conditions and population ageing may also differ by autonomous community, together with other unanalysed factors.

External otitis and ophthalmological complications were the most common complications in HZ hospitalised patients [31]. According to the Centers for Disease Control and Prevention (CDC), postherpetic neuralgia (PHN) is the most common complication of HZ, affecting 10–18% of people with HZ, and the risk seems to increase with age [32]. This was not shown in our results. This may be because immunocompromised status and age are interacting in our population, as we see in Table 3. Furthermore, PHN may not be a severe enough complication to require hospitalisation, with most patients receiving outpatient treatment [33]. Furthermore, PHN usually appears 90 days or more after rash onset, and it is likely that most of these hospitalisations occurred at an earlier stage, so it was not even possible to diagnose PHN at that time [34].

Diabetes mellitus (DM), leukaemia/lymphoma, and chronic obstructive pulmonary disease (COPD), were the most frequent medical conditions in HZ-related hospitalisations. It is well known that immunocompromising conditions such as DM and malignancies result in decreased specific cell-mediated immunity that increases the risk of viral infections, such as varicella zoster [35]. Accumulating evidence also suggests that DM represents an important risk factor for HZ (and its complications) due to attenuated cell-mediated immunity, phagocytosis and opsonisation in this population [36]. Regarding malignancies, we know that hematologic cancer patients develop HZ more quickly following their cancer diagnosis [37,38]. There is also evidence that autoimmunity plays a role in the pathogenesis of COPD, and that the risk of exacerbations of COPD is increased during an HZ episode, particularly in those using oral steroids [39].

In our study both, sex (male) and the presence of comorbidities, increased the risk of hospital readmission in the first month after discharge, while increasing age was associated with a lower risk of readmission, with and without adjustment of other variables. Being female seems to be an independent risk factor for HZ [40], but this relationship is not clear when assessing HZ severity [18]. Several studies have identified other risk factors associated with reactivation of HZ, mainly related to a decrease in T-cell immunity, such as ageing and immunosuppression, but also to family history or stress [41]. We know that HZ reactivation is common in immunocompromised patients, although a considerable amount of heterogeneity is found in the literature [35]. Cell-mediated immunity declines over time due to immunosenescence, but it is normally boosted by exogenous and endogenous methods, thereby limiting varicella zoster virus (VZV)’s ability to reactivate and cause HZ [22,23,37]. Therefore, a more elevated risk in older patients is likely, although younger patients with more severe diseases and/or treatments are more prone to being readmitted after discharge. Human immunodeficiency virus/AIDS was strongly associated with an increased risk of HZ some decades ago, but more recent studies have detected lower incidence rates than previously, showing that appropriate antiretroviral therapy is an important protective factor for HZ [20].

Regarding death, male sex and the presence of comorbidities (except for COPD, asthma and solid organ transplantation) were independent predictors of a higher risk of mortality. Age interacted with other factors; mortality risk was higher at an older age but lower in older age groups once adjusted for comorbidities and sex. Although age seems to be the most important risk factor for the development of HZ, an immunocompromised state at the onset of HZ may have a significantly higher rate of complications [27,42,43], which would support the risk groups being included in the current vaccination policies. As pointed out above, underlying mechanisms contributing to these phenomena may be immunosenescence, and a higher presence of some haematological neoplasms at a lower age [22,37].

Our study presents some limitations associated with the use of hospitalisation records: under-reporting of other existing conditions, possible diagnostic errors, mismatch between ICD codes and the most common medical conditions according to the literature, or difficulties in determining the main cause of death when several disease processes are involved. Moreover, hospital discharge records do not include the cases managed in an outpatient setting. The CMBD system does not provide information about the laboratory tests used for HZ diagnosis, which might impair the quality of the data.

### Conclusions

Our report highlights that HZ is an important public health problem in Spain that needs to be prioritised, especially due to its increasing temporal trend, association with ageing and severity in certain risk groups. We have also shown that the groups included in the current HZ vaccination policies represent precisely the ones with a greater risk of readmission and of fatal outcome. The gradual implementation of HZ vaccination will most probably change the local epidemiology of this disease, as has already occurred in the US [26]. Here, we have established the base for further assessment, as well as provided relevant and innovative information on HZ clinical aspects.

## Supplementary Data

120000 Supplementary Material
