# Authors’ correction for Euro Surveill. 2023;28(8)

**DOI:** 10.2807/1560-7917.ES.2023.28.10.230309c1

**Published:** 2023-03-09

**Authors:** Eurosurveillance editorial team

**Affiliations:** 1 European Centre for Disease Prevention and Control (ECDC), Stockholm, Sweden

In the article ‘Epidemiology of Herpes Zoster in the pre-vaccination era: establishing the baseline for vaccination programme’s impact in Spain’ by Risco Risco et al., published on 23 February 2023, the name of Noemí Lopez-Perea was erroneously written as Noemí Lopez-Parea. This error was corrected on 7 Mar 2023 upon the request of the authors.

