# Supplementary material for: Epidemiology of Herpes Zoster in the pre-vaccination era: establishing the baseline for vaccination programme’s impact in Spain
Source: Euro Surveill. 2023 Feb 23;28(8):2200390. doi: 10.2807/1560-7917.ES.2023.28.8.2200390 (PMC9951257; doi:10.2807/1560-7917.ES.2023.28.8.2200390)
Supplement: Supplementary Material [file 22-00390_HERRADOR_Supplementary_Table_S1.pdf]

This supplementary material is hosted by *Eurosurveillance* as supporting information alongside the article 'Epidemiology of Herpes Zoster in the pre-vaccination era: establishing the baseline for vaccination programme's impact in Spain', on behalf of the authors, who remain responsible for the accuracy and appropriateness of the content. The same standards for ethics, copyright, attributions and permissions as for the article apply. Supplements are not edited by *Eurosurveillance* and the journal is not responsible for the maintenance of any links or email addresses provided therein

Supplementary Table S1. IC-9-CM and IC-10-CM codes for Herpes Zoster and related clinical conditions

**Herpes zoster, HZ complications and related clinical conditions**	**IC-9-CM code**	**IC-10-CM code**
Non complicated form	053.9	B02.9
**Herpes zoster complications**		
External otitis	053.71	B02.8
Ophthalmological complications	053.2	B02.30-34, B02.39
Central nervous system HZ or meningitis HZ	053.0-1	B02.0-1, B02.29
Post herpetic neuralgia	053.12-13	B02.2-3
Disseminated HZ	053.8	B02.7
**Underlying and related clinical conditions**		
Diabetes mellitus	250	E10-11
Asthma	493	J45
Rheumatologic disorders	710, 714	L93, M05-6, M08, M31-36
Chronic obstructive pulmonary disease	491-2	J41, J43, J44
Chronic kidney disease	585	N18
HIV infection	042	B20
Solid organ transplantation	V42 (except V42.81, V42.82) E878	Z94 (except Z94.81-82, Z94.84)
Haematopoietic progenitor cell transplantation	V42.81, V41.82	Z94.81-Z94.84
Leukaemia or Lymphoma	200-208	C81-96
Solid malignant neoplasm	140–209	C, except C81-96

"This supplementary material is hosted by Eurosurveillance as supporting information alongside the article Epidemiology of Herpes Zoster in the pre-vaccination era: establishing the baseline for vaccination programme´s impact in Spain, on behalf of the authors, who remain responsible for the accuracy and appropriateness of the content. The same standards for ethics, copyright, attributions and permissions as for the article apply. Supplements are not edited by Eurosurveillance and the journal is not responsible for the maintenance of any links or email addresses provided therein."
